# Gender composition and wage gaps in the Canadian health policy research workforce in comparative perspective

**DOI:** 10.1186/s12960-022-00774-5

**Published:** 2022-11-07

**Authors:** Neeru Gupta, Sarah Ann Balcom, Paramdeep Singh

**Affiliations:** 1 Department of Sociology, University of New Brunswick, PO Box 4400, Fredericton, NB E3B 5A3 Canada; 2 Faculty of Nursing, University of New Brunswick, Fredericton, Canada; 3 Institute for Research, Data and Training (IRDT), University of New Brunswick, Fredericton, Canada

**Keywords:** Health workforce, Health occupations, Health policy research, Professional labour markets, Wage differentials, Gender inequality, Gender wage gap, Statistics and numerical data

## Abstract

**Background:**

Gendered challenges have been shown to persist among health practitioners in countries at all levels of development. Less is known about non-clinical professionals, that is, those who do not deliver services directly but are essential to health systems performance, such as health policy researchers. This national observational study examined gender occupational segregation and wage gaps in the Canadian health policy research workforce using a cross-domain comparative labour market analysis approach.

**Methods:**

Sourcing data from the 2016 population census, we applied linear regression and Oaxaca–Blinder decomposition techniques to assess wage differentials by sex, traditional human capital measures (e.g., age, education, place of work), and social identity variables intersecting with gender (household head, childcare, migrant status) among health policy researchers aged 25–54. We compared the gender composition and wage gap with seven non-health policy and programme domains, as mapped under the national occupational classification by similarity in the types of work performed.

**Results:**

The health policy research workforce (*N* = 19 955) was characterized by gender segregation: 74% women, compared with 58% women among non-health policy research occupations (*N* = 102 555). Women health policy researchers earned on average 4.8% (95% CI 1.5‒8.0%) less than men after adjusting for other professional and personal variables. This gap was wider than among education policy researchers with similar gender composition (75% women; adjusted wage gap of 2.6%). Wages among health policy researchers were 21.1% (95% CI 19.4‒22.8%) lower than their counterparts in the male-dominated economics policy domain, all else being equal. Overall, women’s earnings averaged 3.2% lower than men’s due to factors that remained unexplained by policy domain or other measured predictors.

**Conclusions:**

This investigation found that the gender inequalities already widely seen among clinical practitioners are replicated among health policy researchers, potentially hindering the competitiveness of the health sector for attracting and retaining talent. Our findings suggest intersectoral actions are necessary to tackle wage gaps and devaluation of female-dominated health professions. Accountability for gender equity in health must extend to the professionals tasked with conducting equity-informative health policy research.

## Introduction

An estimated 67% of the global health workforce are women, but persistent gender challenges mean the promise of “equal pay” for “equal work” remains elusive [1]. The World Health Organization (WHO) advocates for the need to consider gender as a potential driver of social inequalities among human resources for health (HRH)—and therefore to systematically incorporate gender issues in health labour market analyses as a means to ensure the full, effective, and equitable participation of women and men in a high performing health workforce to meet current and future population health needs [2]. Traditionally gendered divisions of labour and social norms may lead to certain professions, including female-dominated nursing and caregiving occupations, being given lower social value and lower pay [3–6]. Income inequalities and other gender-related problems have also been widely documented among women in traditionally male-dominated health occupations, such as medicine [4, 7–9]. It is increasingly recognized that health systems should be held accountable to sociocultural imbalances and establish equity benchmarks, but gender considerations often remain neglected in health systems and health workforce research [10–12]. In a landmark 2019 report, the WHO identified four thematic areas with major gaps in the global HRH data and literature to support gender inclusiveness and equity: occupational segregation, gender pay gaps, decent work, and leadership [13]. An example of such information deficiencies may include gender wage gaps experienced by the very professionals involved in the design and application of gender-responsive health policy.

Differences in compensation for human capital endowments, such as professional qualifications, may help explain gender wage gaps; nonetheless, research persistently indicates simply being a women or employed in an occupation with skewed gender composition are major contributors to wage differentials [14]. The causes of gender occupational segregation and wage gaps among HRH have been linked sociologically and psychologically to historical idealizations defined by and for professional men, who were expected to be authoritative, rational, and committed to their jobs and earning more money; women were typically seen as lacking such traits and thus less committed to employment and becoming professional leaders [3, 5]. Despite social change and women’s increased participation in higher education, the influx of women into previously male-dominated professions has not eliminated differential treatment by gender and may render some jobs less rewarding to men [3, 5, 9]. In many countries traditional gendered hierarchies remain entrenched within health system institutional practices, such that social biases continue to shape compensation policies, professional development opportunities, and productivity evaluations [4, 14, 15]. The result, as argued by Adams, is that women tend to maintain their presence in historically female-dominated occupations (such as nursing), some feminizing professions are internally devalued (e.g., paediatricians), while many higher-paying professions continue to be male-dominated (e.g., surgical specialists) [3]. Such gendered wage relativities may exacerbate HRH shortages if the (female-dominated) health sector is perceived to offer less attractive career trajectories than other sectors (e.g., male-dominated, higher-paying economics and business professions) [5, 6].

Numerous observational studies have found significantly lower earnings for women health workers and feminizing health occupations within and across countries and over time. In an international study of health worker wages from 21 countries, Boniol et al. reported that women health workers earned 28% less than men on average; after adjusting for key labour market variables, a gap of 11% remained for women and men with similar occupations and working hours [1]. A cross-national analysis of health workforce remuneration associated increasing shares of women participating in a given health occupation with a decrease in its wage rank [16]. Based on a time-trend analysis of sex-disaggregated survey data from 25 countries, Shannon et al. suggested that the gender wage gap was widening with increasing feminization of the health labour market, particularly in selected lower- and upper-middle-income countries [17]. In one high-income setting, an analysis of administrative data from the Canadian province of British Columbia indicated significantly lower earnings among women physicians compared with men after adjusting for patient contacts and other factors [18]. In Iran, Rad et al. found that women physician’s salaries averaged 30% less than men’s, with 7% of the wage disparity remaining unexplained after controlling for speciality, work experience, and other measured confounders [19]. A 19% unexplained component to the raw gender wage gap was found among health professionals in Australia, after decomposing gender differences in job characteristics and other human capital factors [14]. From a cross-sector comparative perspective, the evidence is inconclusive as to whether the health sector is more unequal than non-health sectors. In one analysis of data from five countries, gender wage gaps were found to be less pronounced among (male-dominated) science professionals than (female-dominated) nursing professionals, while the association was inconsistent in relation to (female-dominated) education occupations [20].

While wages are central to HRH recruitment and retention, a lack of comprehensive wage information covering the broad scope of health occupations has constrained health workforce strengthening [16]. Studies on the gender wage gap typically focus on service providers. However, an estimated one-third of the global health workforce are composed of health management and support personnel, that is, those who do not provide direct patient care but are essential to the performance of health systems [21]. This may include professionals with clinical backgrounds working outside the healthcare sector. For example, data on physicians in academic institutions in the United States have told of pervasive gender-based salary disparities among early-career and mid-career professionals, even after adjustment for factors such as specialty and academic rank [22, 23]. This wide-ranging category of HRH may also include individuals with non-clinical professional skills employed in the health system, such as those whose core responsibilities involve health policy and programme research and development. Their labour market profile is much less known.

In Canada, women are overrepresented in health employment and continue to increase their shares in many professions requiring a university degree, including family medicine and health policy research [24]. We are unaware of any studies in Canada (or elsewhere) explicitly addressing gender-related wage gaps in the health policy research workforce. To build the evidence base on wage conditions among health policy researchers, this study investigates the gender composition and relative wages among health policy researchers in the Canadian national context. We address whether gender occupational segregation and wage gaps are issues within the health policy research workforce and compared with other non-health policy domains. We use data from the 2016 Canadian population census to assess and decompose wage disparities among policy researchers in health and other traditionally female-dominated sectors in relation to selected traditionally male-dominated sectors. Econometric decomposition analysis is applied to estimate the explained portion of observed wage differences between women and men as well as any residual “unexplained” component, the latter being commonly interpreted as a statistical measure of gender discrimination [5, 14, 25].

## Methods and materials

### Study design and target population

Our data source was the Canadian Population Census, conducted quinquennially by Statistics Canada. The census entails a complete enumeration of the population; we used microdata from the 2016 mandatory long-form questionnaire, which was distributed to a 25% sample of all households and collected detailed sociodemographic and labour market information. We limited the analysis to employed persons in the prime working ages of 25 to 54 years, with a bachelor’s degree or higher educational attainment, and who reported having earned wages or salaries in the year preceding the census (i.e. the 2015 calendar year). The earnings data were captured from integrated administrative income tax and benefits records [26]. The response rates for the 2016 census were 97.8% for the long-form questionnaire overall and 97.1% for occupational earnings among long-form respondents [26, 27].

The health policy research workforce was identified according to the systematic taxonomy of the National Occupational Classification (NOC code 4165) [28]. This occupation is described as persons who specialize in research and analysis to support the development, administration, and assessment of government and non-government health policies, programmes, and standards as the main duties of their job. These positions generally require a post-secondary degree in health science, hospital administration, social science, or another related field. Managerial positions, clinical service providers, and academic researchers are excluded (classified elsewhere).

We also compared wages among health policy researchers with their counterparts in other socioeconomic and scientific domains. As a tool for employment equity monitoring, the NOC structures occupations based on similarities in work duties, responsibilities, and requirements. We evaluated a total of eight occupations within the minor group “Policy and programme researchers” (hierarchically arranged within NOC code 416) (Table 1) [28]. This category includes policy research professionals in a variety of social, legal, community, and government services. Given the similarities of skills and work usually performed as well as the overlapping educational routes for entering employment in these occupations, wage structures are not generally expected to vary significantly in the absence of other structural or normative criteria, such as gendered valuation.

**Table 1:** Policy research domains distinguished in the 2016 National Occupational Classification (NOC)

NOC code	Occupational title	Description
4161	Natural and applied science policy researchers	Conduct research, prepare reports, provide consultative advice, and administer and evaluate policies and programmes in areas related to the natural and applied sciences (e.g., natural resources policy analyst, transportation safety analyst)
4162	Economic policy researchers	Conduct research, analyse data and information, and advise on matters to resolve economic and business problems (e.g., economic policy analyst, labour economist)
4163	Business development officers	Conduct research, formulate policies, and evaluate programmes to promote industrial and commercial business investment or tourism (e.g., market researcher, regional development analyst)
4164	Social policy researchers	Conduct research, assess social legislation, and administer and evaluate policies and programmes in areas such as employment, immigration, law enforcement, human rights, and housing (e.g., community social development officer, employment equity policy consultant)
4165	Health policy researchers	Conduct research, produce reports, and administer and evaluate health care policies and programmes (e.g., health policy research analyst, health services researcher, nursing homes policy development officer)
4166	Education policy researchers	Conduct research, produce reports, and administer and evaluate elementary, secondary, and post-secondary education policies and curriculum programmes (e.g., education policy officer, curriculum developer)
4167	Recreation, sports, and fitness policy researchers	Conduct research and provide consultative advice on policies and programmes related to recreation, sports, and physical fitness (e.g., recreologist, sports policy analyst, fitness policy analyst)
4168	Programme officers unique to government	Provide research-based advice on the social, economic, and political effects of public policies and issues on government institutions (e.g., intergovernmental affairs officer, foreign service officer)

Source: Adapted from Employment and Social Development Canada (ESDC) and Statistics Canada

### Analytical methods

Descriptive, bivariate (correlation), and multivariate (regression-based) analyses were conducted to assess and decompose gender differences in wages within and across the eight groups of policy researchers. The outcome of interest was individuals’ annual gross wages and other employment remuneration (i.e. before deductions for income taxes, pension plan contributions, and other social premiums), as measured in Canadian dollars.

The key predictor, gender, was captured as female or male (based on the dichotomous response options available in the 2016 census questionnaire). We further considered a number of labour market variables widely postulated as influencing occupational earnings: age (grouped into three categories across the core working life span: early career 25–34 years, mid-career 35–44 years, and 45–54 years), educational attainment (at most bachelor’s degree versus graduate-level studies), full-time versus part-time work, class of worker (whether the person was an employee or in self-employment), sector of work (whether the person was working in public administration or elsewhere). Other social variables commonly regarded to intersect with gender were included in the analysis, including designation as the primary household maintainer (sometimes referred to as the household head), marital status (whether or not the person was living in a marital or common-law union situation), child presence (whether or not the household included any children), and adult migrant status (whether or not the person had immigrated to Canada in adulthood, i.e. above age 19). The province or region of residence was also included to control for observed and unobserved influences on wage variance.

Following a log-transformation of the wages variable to account for data skewness, we employed simple linear regressions to assess the (unadjusted) gender wage gap, and then multiple linear regressions to assess the independent associations of gender and other labour market, social, and residential predictors on differences in occupational earnings. We ran separate models for each occupation, and then one pooled model including all eight occupations combined.

Lastly, we examined the difference in mean (logged) wages between men and women using the Oaxaca–Blinder decomposition method to recognize which average characteristics of men and women “explained” a portion of the wage gap, and what was left “unexplained” [29, 30]. Widely applied in investigations of social inequalities in health and labour outcomes, the unexplained component of the linear regression-based decomposition is often attributed to discrimination, i.e. a situation where persons with identical capacities and characteristics receive different benefits compared with others [25, 31]. The analysis was conducted using the Stata statistical software [32].

The de-identified census microdata used in this study were accessed in the secure computing facilities of the Statistics Canada Research Data Centre at the University of New Brunswick (Fredericton, Canada). Person-level bootstrapped sampling weights were applied to ensure population representation of the parameters and robust 95% confidence intervals (CIs). Population counts were rounded and all statistical outputs were subject to risk-based confidentiality vetting in respect of Statistics Canada data privacy protocols.

## Results

### Descriptive analysis

According to the 2016 census, 122 510 Canadians aged 25–54 were employed in a policy research occupation, with this workforce characterized as predominantly female (61% women) (Table 2). Specifically, one in six (16%) were working in health policy research, a domain characterized by more pronounced gender segregation (74% women). Of the eight policy research occupations under observation, only the economics policy research workforce was male-dominated (44% women). The remaining occupations under observation tallied 53‒75% women.

**Table 2:** Gender distribution and wage conditions among health and non-health policy researchers aged 25–54

Policy domain	Workforce population		Percent women	Mean annual wage (CAD $)		Gender earnings ratio
	Number	Share (%)		Female	Male	
Natural and applied science	15 575	12.7	53.2	71 206	86 771	0.82
Economics	12 140	9.9	43.7	82 714	109 818	0.75
Business development	30 335	24.8	53.5	66 047	92 189	0.72
Social	19 210	15.7	67.2	63 343	71 759	0.88
Health	19 955	16.3	74.1	65 895	75 271	0.88
Education	13 115	10.7	74.9	61 279	69 926	0.88
Recreation, sports, and fitness	4525	3.7	66.0	47 922	52 532	0.91
Government programmes	7655	6.2	57.4	70 694	82 253	0.86
Total	122 510	100.0	61.0	66 229	85 869	0.77

The gender earnings ratio refers to women’s mean annual wage as a percentage of men’s. Policy domains categorized by the 2016 National Occupational Classification

Source: 2016 Canadian Population Census (authors’ calculations; data weighted for population representation)

All eight occupations were characterized with lower average annual earnings among women than men, despite similarities in job duties and working conditions. Women in the health policy research workforce earned an average of 88 cents for every dollar earned by men (Table 2). Across the other occupations, the gender earnings ratio ranged from 72 to 91 cents to the dollar. Occupations in traditionally male-dominated sectors (notably, the economics, natural and applied science, and business development policy domains) tended to offer higher average levels of remuneration than occupations in traditionally female-dominated sectors (including the health, education, social, and recreation policy domains). The higher-paying occupations were also characterized with wider gender earnings ratios (72‒82 cents to the dollar) than their counterparts in traditionally female-dominated sectors (88‒91 cents to the dollar).

**Fig. 1:**
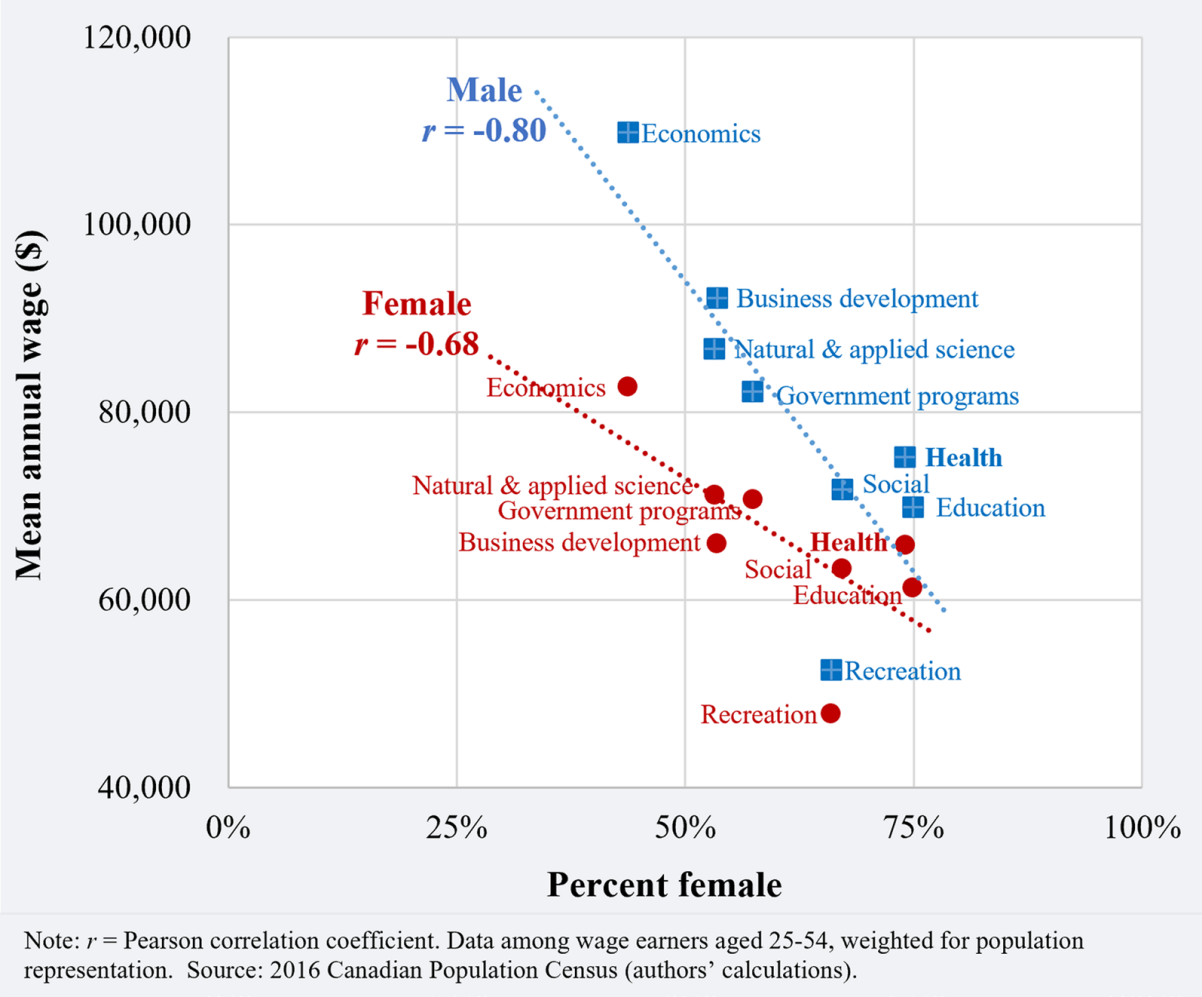
Mean annual wage by percent female among health and non-health policy researchers, according to policy domain

Across occupations, having a higher share of women was correlated with lower mean wages among women (*r* = − 0.68) (Fig. 1). The negative correlation of occupational feminization was even stronger in terms of dropping mean wages among men (*r* = − 0.80).

**Fig. 2:**
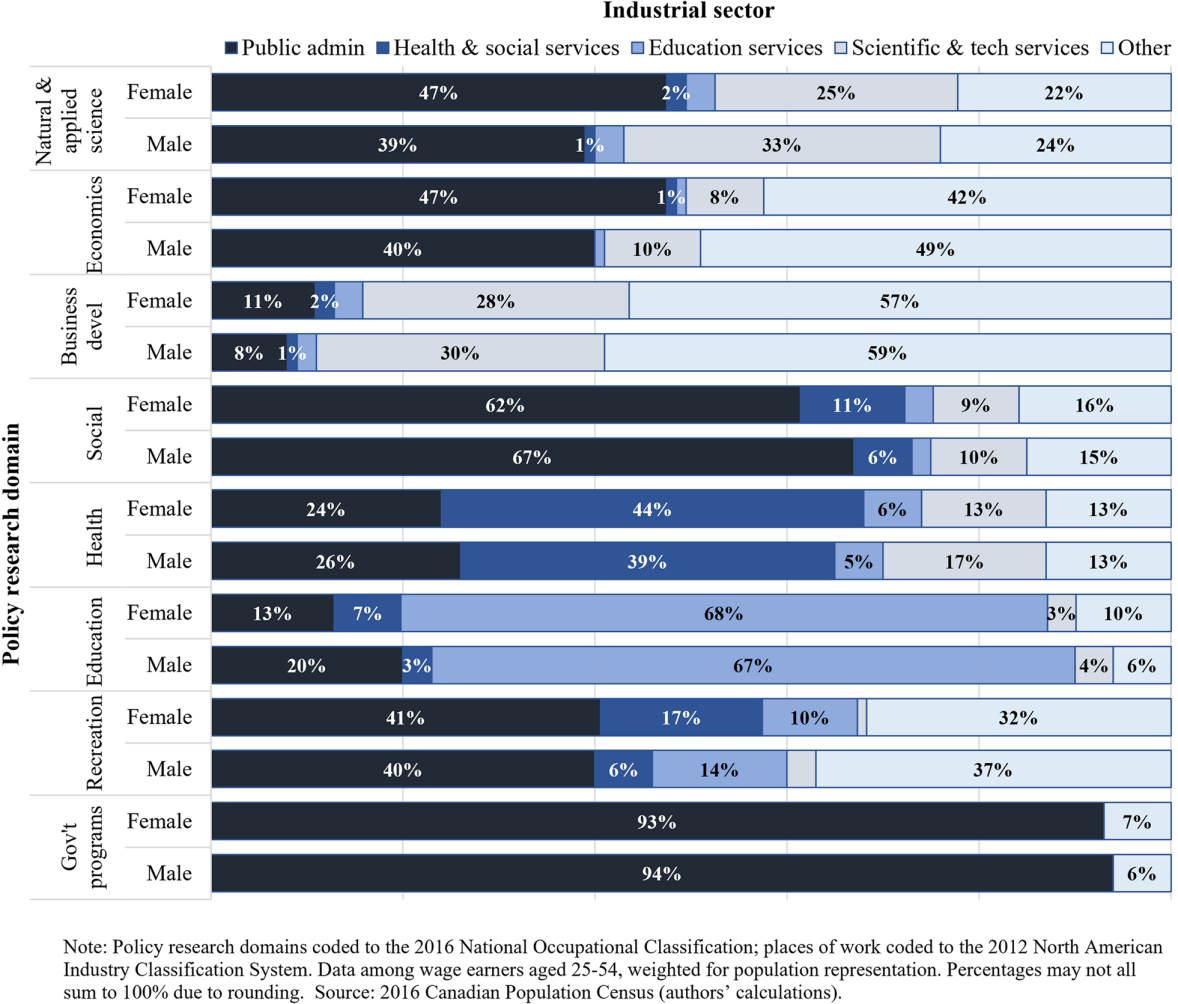
Percentage distribution by place of work among health and non-health policy researchers, according to policy domain

Health policy researchers were primary employed in healthcare and social assistance establishments (females: 44%; males: 39%) and in public administration (females: 24%; males: 26%), although not exclusively so (Fig. 2). Non-negligible numbers were engaged in educational services and in other scientific and technical services. Conversely, healthcare and social assistance establishments engaged large numbers of recreation policy researchers (females: 17%; males: 6%) and social policy researchers (females: 11%; males: 6%). In other words, the boundaries of the health system were not easily delineated by any given policy research domain.

The age structure of the health policy research workforce differed little by gender (Table 3). In contrast, women policy researchers in natural and applied science domains and in business development domains tended to be younger than men, that is, more often in the 25–34 years age group—a reflection of the feminization of sectors where women have been traditionally underrepresented. Regarding other key labour market variables, women health policy researchers were characterized less often than men with a graduate-level qualification (58% versus 61%) and more often in part-time work (11% versus 8%). In terms of social identity variables, women health policy researchers reported significantly less often than men as being the primary household maintainer (50% versus 72%), yet more often residing in a household with children present (53% versus 48%). Women were also less likely than men to have been adult migrants to Canada (15% versus 26%).

**Table 3:** Percentage distribution of the health and non-health policy research workforces by sociodemographic and labour market characteristics, according to gender

Characteristic	(1) Natural & applied science		(2) Economics		(3) Business development		(4) Social		(5) Health		(6) Education		(7) Recreation		(8) Government programmes	
	Female (%)	Male (%)	Female (%)	Male (%)	Female (%)	Male (%)	Female (%)	Male (%)	Female (%)	Male (%)	Female (%)	Male (%)	Female (%)	Male (%)	Female (%)	Male (%)
Age group																
25–34 years	38*	31	39	40	49*	43	39*	34	38	37	28	29	51	52	32*	26
35–44 years	39	40	36	35	32	32	37	38	36	35	38	37	28	30	41	39
45–54 years	22*	29	25	25	20*	24	25*	28	26	28	34	34	20	18	27*	35
Educational attainment																
Bachelor’s degree	46	48	44*	40	70*	66	50*	47	42*	39	50*	44	80	79	57	56
Graduate level	54	52	56*	60	30*	34	50*	53	58*	61	50*	56	20	21	43	44
Work status																
Full time	91*	96	96*	98	91*	96	92*	96	89*	93	87*	92	82*	87	89*	94
Part time	9*	4	5*	2	9*	4	8*	4	11*	8	13*	8	18*	10	11*	6
Class of worker																
Employee	96*	94	99	98	93	93	97	96	98	97	97*	95	98*	96	100	100
Self-employed	4*	6	1	2	7	7	3	4	2	3	3*	5	2*	4	0	0
Primary household maintainer																
Primary	56*	70	54*	76	48*	68	56*	72	50*	72	51*	72	48*	61	53*	74
Not primary	44*	30	46*	24	52*	32	44*	28	50*	28	49*	28	52*	39	47*	26
Marital status																
With spouse/partner	71*	75	67	68	64*	68	66*	72	69	69	70*	73	67	64	68*	72
No spouse/partner	29*	25	33	32	36*	32	34*	28	31	31	30*	27	33	36	32*	28
Household presence of children																
With children	51*	56	51	47	44*	48	50	50	53*	48	57	54	48*	35	53	53
No children	49*	44	49	53	56*	52	50	49	47*	52	43	46	51*	65	47	47
Adult migrant status																
Immigrated in adulthood	12*	17	28*	24	19*	22	16	17	15*	26	11*	19	7	7	10	11
Not adult migrant	88*	83	72*	76	81*	78	84	83	85*	74	89*	81	92	93	90	89
Province or region of residence																
Atlantic region	5	5	3	3	4	4	6	4	5	4	7	8	5	8	5	5
Quebec	21	22	20	22	22*	15	23*	25	2	20	27	26	22	22	23	24
Ontario	42	39	58	56	49*	51	45*	47	52	50	37	36	37	38	53	53
Prairies region	18	20	10	12	12*	16	14*	13	14	15	16	16	18	17	10	10
British Columbia	12	12	8	6	13	13	11	10	10	11	12	12	17	15	8	7
Northern region	2	1	0	0	0	0	1	1	1	0	1	1	1	0	1	1

*p < 0.05 (statistically different from the male reference group). Not adult migrant: Canadian-born or immigrated before age 20. Atlantic provinces: Newfoundland & Labrador, Nova Scotia, Prince Edward Island, and New Brunswick; Prairie provinces: Manitoba, Saskatchewan, and Alberta; Northern territories: Yukon, Northwest Territories, and Nunavut

Source: 2016 Canadian Population Census (authors’ calculations; data weighted for population representation)

### Bivariate analysis of wage differentials by gender

**Fig. 3:**
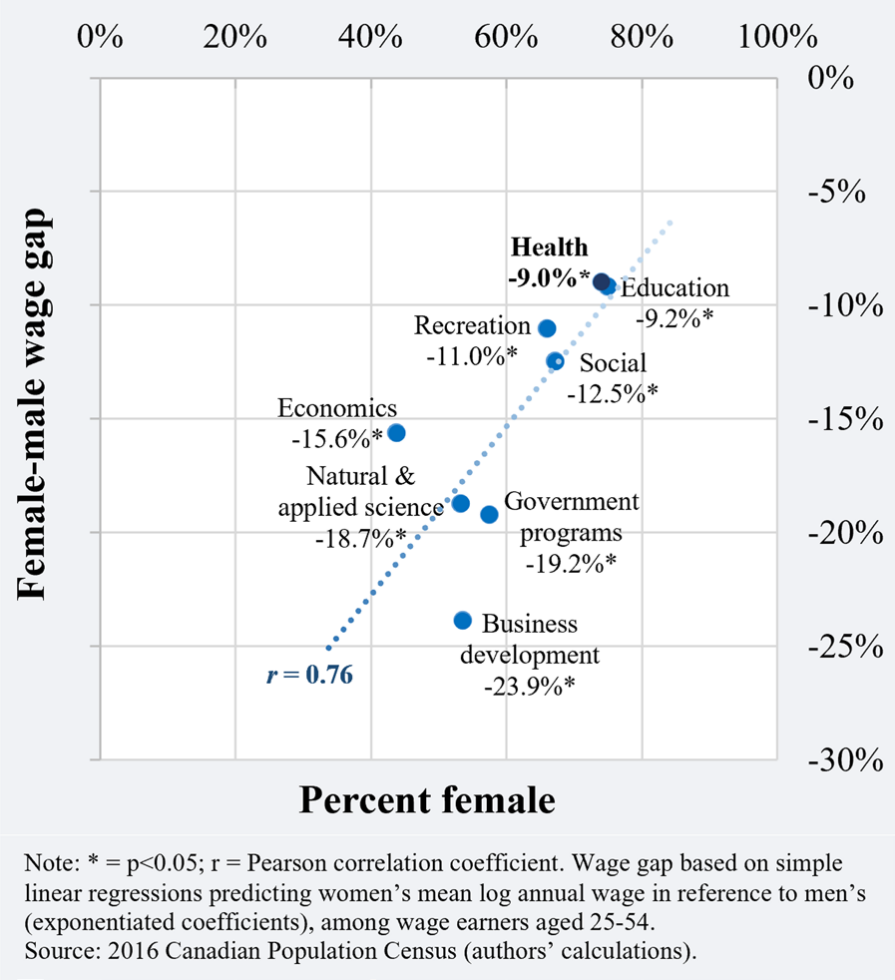
Female‒male wage gap by percent female among health and non-health policy researchers, according to policy domain

Based on the simple linear regression model, women health policy researchers were found to have earned 9.0% (95% CI 5.1‒12.7%; *p* < 0.05) less than men. This was the narrowest (unadjusted) female‒male wage gap among the eight occupations under observation, which otherwise ranged between 9.2% (among education policy researchers) and 23.9% (among business development policy researchers) (Fig. 3). The bivariate analysis of the policy research workforce also confirmed a strong positive correlation between the degree of occupational feminization and the size of the gender wage gap (*r* = 0.76).

### Multivariate and decomposition analyses of the gender wage gap

The multivariable linear regression analysis upheld the evidence of a significant gender wage gap in the health policy research workforce, with women earning 4.8% (95% CI 1.5‒8.0%) less than men, after adjusting for other labour, social, and residential characteristics (Table 4, model 5). Those in their early career stage (aged 25–34) tended to earn less than their more established colleagues, all else being equal, as did those who had immigrated to the country in adulthood compared with their counterparts who were native-born or who had migrated in childhood or adolescence (i.e. prior to exposure to advanced education and labour market access).

**Table 4:** Coefficients (and 95% confidence intervals) from the linear regression models for predictors of annual wages among health and non-health policy researchers

Predictor variable	(1)	(2)	(3)	(4)	(5)	(6)	(7)	(8)
	Natural and applied science	Economics	Business development	Social	Health	Education	Recreation	Government programmes
Gender								
Female (ref: Male)	− 0.111*	− 0.084*	− 0.123*	− 0.040*	− 0.048*	− 0.027	− 0.069*	0.019
	(− 0.140 to − 0.081)	(− 0.112 to − 0.054)	(− 0.144 to − 0.101)	(− 0.063 to − 0.017)	(− 0.080 to − 0.015)	(− 0.068 to 0.018)	(− 0.131 to − 0.003)	(− 0.031 to 0.073)
Age group								
25–34 years (ref: 35–44 years)	− 0.377*	− 0.298*	− 0.310*	− 0.283*	− 0.313*	− 0.308*	− 0.324*	− 0.213*
	(− 0.401 to − 0.351)	(− 0.319 to − 0.276)	(− 0.330 to − 0.289)	(− 0.304 to − 0.262)	(− 0.335 to − 0.29)	(− 0.338 to − 0.277)	(− 0.373 to − 0.270)	(− 0.267 to − 0.155)
45–54 years (ref: 35–44 years)	0.090*	0.180*	0.210*	0.180*	0.210*	0.190*	0.080	0.027
	(0.051 to 0.140)	(0.145 to 0.219)	(0.168 to 0.260)	(0.145 to 0.210)	(0.170 to 0.246)	(0.153 to 0.234)	(− 0.035 to 0.202)	(− 0.052 to 0.091)
Educational attainment								
Graduate level (ref: At most Bachelor's degree)	0.022	0.202*	0.103*	0.065*	0.063*	0.091*	− 0.064	0.147*
	(− 0.005 to 0.051)	(0.164 to 0.241)	(0.073 to 0.133)	(0.037 to 0.093)	(0.031 to 0.095)	(0.058 to 0.125)	(− 0.140 to 0.019)	(0.091 to 0.207)
Work status								
Full time (ref: Part time)	2.062*	2.808*	2.662*	1.889*	2.449*	2.337*	1.895*	17.375*
	(1.765 to 2.387)	(2.177 to 3.563)	(2.377 to 2.971)	(1.654 to 2.146)	(2.225 to 2.691)	(2.043 to 2.658)	(1.565 to 2.271)	(14.196 to 21.242)
Industrial sector								
Public administration (ref: Other)	0.037*	− 0.148*	− 0.001	0.322*	0.148*	0.156*	0.387*	− 0.068
	(0.007 to 0.068)	(− 0.180 to − 0.131)	(− 0.032 to 0.033)	(0.281 to 0.363)	(0.120 to 0.178)	(0.124 to 0.190)	(0.307 to 0.471)	(− 0.160 to 0.015)
Household presence of children								
Children present (ref: No children)	− 0.0222	0.059*	− 0.010	0.006	− 0.029	− 0.012	− 0.030	0.106*
	(− 0.058 to 0.016)	(0.019 to 0.101)	(− 0.038 to 0.020)	(− 0.021 to 0.034)	(− 0.059 to 0.003)	(− 0.044 to 0.021)	(− 0.099 to 0.046)	(0.047 to 0.169)
Adult migrant status								
Immigrated in adulthood (ref: Not adult migrant)	− 0.204*	− 0.234*	− 0.317*	− 0.264*	− 0.250*	− 0.295*	− 0.252*	− 0.258*
	(− 0.244 to − 0.162)	(− 0.265 to − 0.202)	(− 0.343 to − 0.289)	(− 0.297 to − 0.231)	(− 0.281 to − 0.218)	(− 0.341 to − 0.246)	(− 0.386 to − 0.088)	(− 0.340 to − 0.166)

*p < 0.05; ref: reference category. Results based on multiple linear regressions predicting mean log annual wage, among wage earners aged 25–54 (exponentiated parameters). Models further adjusted for class of worker (except among policy researchers for programmes unique to government), primary household maintainer status, marital status, and province/region of residence

Source: 2016 Canadian Population Census (authors’ calculations)

Across non-health policy research occupations, the gender wage gap held as significant for five other domains: women’s earnings averaged from 4.0% less (among social policy researchers) to 12.3% less (among business development policy researchers) than men’s earnings (Table 4). No discernible gender-based wage gaps were found for policy researchers in government programmes and in education domains, among whom any raw wages differentials were largely attributable to age, graduate-level educational attainment, and adult migrant status.

In a regression model pooling all eight policy research domains together, the seven female-dominated occupations were each found to pay significantly less on average than economics policy research (i.e. the sole male-dominated occupation under observation), all else being equal (not shown). In particular, the mean annual wage among health policy researchers was 21.1% (95% CI 19.4‒22.8%) lower than their counterparts in economics policy research. In relation to economics policy researchers, wages averaged from 15.4% less (among business development policy researchers) to 36.2% less (among recreation policy researchers). The overall gender wage gap held as significant, with the mean earnings of women assessed at 8.1% (95% CI 6.9‒9.2%) lower than men, regardless of policy domain or other professional or personal characteristics.

The decomposition analysis indicated that, as could be expected, differences between women and men in educational attainment and other traditional human capital variables accounted for much (27%) of the gender wage gap in the policy research workforce (Table 5). However, 15% of the wage differential was attributable to occupational differences, i.e. by the domain of policy and programme research, distinctly from other labour characteristics. The gender wage gap was less pronounced in health policy research compared with the (better-paid) economics policy domain. Age differences between women and men accounted for 6% of the wage differential and differences in social identity characteristics accounted for 10% of the differential. After decomposing gender differences in professional wages, a significant 40% of the gap remained unexplained by the measured predictors.

**Table 5:** Explained and unexplained components of the female‒male wage differential in the policy research workforce (eight pooled policy domains)

Component	Coefficient (% of gap)
Explained	0.1271* (60%)
Occupation	0.0319* (15%)
Health policy research	− 0.0018* (− 6%)
Economics policy research	0.0185* (58%)
Other non-health policy domains	0.0152* (48%)
Age group	0.0120* (6%)
Other labour characteristics	0.0571* (27%)
Social identity characteristics	0.0211* (10%)
Province/region of residence	0.0050* (2%)
Unexplained	0.0845* (40%)
Total female‒male differential	0.2115* (100%)

*p < 0.05. Components estimated using Oaxaca–Blinder decomposition representing the percent of the total log wage differential explained by gender differences in the measured factors, among wage earners aged 25–54. Policy domains categorized by the 2016 National Occupational Classification

Source: 2016 Canadian Population Census (authors’ calculations)

## Discussion

While several HRH studies have examined the persistence of wage differentials between women and men engaged in clinical services, this inquiry represents the first nationally representative analysis in Canada (or, to our knowledge, anywhere) of gendered wage conditions among health policy researchers. Non-clinical professionals represent a large component of human resources in health systems, and policy researchers play an integral role in the development and monitoring of equity-enhancing government and community health policies and programmes. As such, managing gender disparities in health services delivery requires an understanding of underlying gender issues within the workforce itself tasked with policy-actionable research. Echoing global HRH trends in gender composition, the Canadian health policy research workforce was enumerated as predominantly female (74% women). Perhaps not surprisingly, we found evidence of a significant gender wage gap, with women earning 4.8% (95% CI 1.5‒8.0%) less on average annually than men, after adjusting for age, education, and other labour, social, and residential characteristics.

As examinations of health labour markets are enhanced when placed in a more comprehensive perspective that takes into account other sectors [20], we compared wages among health policy researchers with those for selected non-health policy research occupations. Disconcertingly in terms of the relative competitiveness of the health sector for attracting and retaining talent, results from our pooled cross-domain linear regression presented significantly lower wages among health policy researchers (21% lower; *p* < 0.05) than their counterparts in economics policy research, the only male-dominated occupation under observation (56% men). These results were consistent with research evidence elsewhere of diminishing wage conditions with increasing shares of females in a given occupation. Such findings may express societal devaluation of “women’s work” in the labour market, and replicate and reinforce social perceptions of gendered differences in professional status even for similar types of work [3, 6, 14–16].

Moreover, the cross-domain regression and Oaxaca–Blinder decomposition analyses showed, of the (observed) female‒male wage differential of 8.1% (95% CI 6.9‒9.2%) in the total policy research workforce, 40% remained unexplained by the measured predictors. In other words, women’s earnings averaged around 3.2% less than men’s due to unexplained factors, an outcome that may be attributed, at least in part, to (unobserved) gender discrimination and other sociocultural and economic structures that hinder women’s labour market opportunities. Significant adjusted wage gaps among healthcare workers have been reported across different national income contexts in studies using decomposition techniques, to provide insight on the residual wage gap that cannot be accounted for by differences in women’s and men’s individual characteristics [6, 14, 19]. The unexplained residual of decomposed wage levels is widely postulated in the literature to capture effects of female‒male differences in societal conventions, unconscious bias, self-selection, and other unmeasured processes leading to a systematic, avoidable, and unfair maldistribution of resources and benefits [6, 16, 25, 31]. It may, however, also denote some degree of estimation bias related to variables omitted from the operationalized model. For example, Vecchio et al. attributed a small part of the wage gap in Australia’s health sector to gendered patterns of unpaid overtime [14]; the lack of a question on expected hours of work in our data source precluded the ability to integrate this potential confounder.

The need for intersectoral collaboration and cooperation is accepted internationally as a critical principle for advancing population health and health equity goals [33]. Our analysis underscored that actions for health workforce strengthening and equity cannot be achieved within the health sector alone. Sectors are largely social constructs, and calls for policy integration are increasing [33]. Alignment of labour market approaches should be considered necessary to improve and sustain the efficiency, effectiveness, and inclusiveness of work conditions among women and men with policy research expertise in the health sector and across interdependent sectors.

### Study strengths and limitations

A number of data sources may potentially provide relevant information to support health labour market analyses, each with their strengths and limitations [2]. We used national population census data, which offer the advantage of large sample sizes covering all labour sectors that can be disaggregated by sex and for specific occupations. A limitation to this cross-sectional source is possible selection bias from the inclusion of only those who were currently participating in the policy research workforce. The analysis thus excluded those who may not have entered the paid labour market for parental or other caregiving reasons as well as those with previous policy research experience who may have ascended to higher-paying managerial occupations—both of which may be gendered processes. Unlike many clinical professions, the educational trajectory of health policy researchers can be diverse (e.g., may include public administration or other fields outside of health sciences). As such, we were unable to identify within the available data those who may have acquired qualifications for employment in health policy research but were no longer in the labour force at the time of data capture. We applied different techniques to analyse wage differentials, from inclusion of a sex dummy variable in single regressions to flexible decomposition methods. Similarly to other HRH studies, we did not correct for selectivity bias using complex maximum likelihood models (e.g., the Heckman correction) that might potentially introduce greater uncertainty and heterogeneity to the population sample [14].

Our operational definition of the health policy research workforce may not have covered the gamut of personnel contributing to the development, administration, and evaluation of policies and programmes, such as public health epidemiologists or academic-based scientists—who are grouped elsewhere in the Canadian occupational classification with other professionals with similar skill levels and specializations. That said, the present ability to readily delineate health policy researchers as a statistical unit ensures cross-domain comparability with non-health policy researchers. We thus anticipate this analysis may be reproduced and updated upon release of the 2021 national census microdata, which were not available for research use at the time of this study. Cross-national comparative analyses of the health policy research workforce are still hampered by a lack of alignment in certain concepts with the International Labour Organization’s International Standard Classification of Occupations (ISCO). The latter structurally identifies some policy research occupations; perhaps related to greater emphasis on transferability of skills, there is no direct concordance by policy domain. In particular, the current ISCO version (last updated in 2008, known as ISCO-08) aggregates at the lowest level of classification all those charged with researching and analysing policy options among “Policy administration professionals” (unit group 2422) [34]. Special attention is needed for mapping health policy researchers distinctly from the broader policy research landscape. Harmonizing labour force data based on the place of work would be inadequate. As our results highlighted, including only those working in health services establishments would miss more than half of all health policy researchers based on occupational descriptors.

## Conclusions

Like in many countries and at the international level, Canada’s health policy dialogues are dominated by shortages and imbalances in the health workforce, exacerbated by the Covid-19 pandemic. It is increasingly argued reversing such trends requires investing in female health workers, given that the health sector relies heavily on the recruitment and retention of women [35]. The WHO has long recognized that the people working in planning and setting directions for health systems are indispensable, but often overlooked in HRH data and discussions [21]. Addressing gender inequity and wage differentials across the health policy pipeline is one important element, including among those tasked with bridging information from clinical and community health programmes to evidence-based advice for decision-making. Devaluation of women’s contributions to the performance of health systems should be viewed as an ongoing crisis [9], although denial among researchers and the wider public of persistent gender inequality in the realm of work is widespread [5]. As the present findings need to be tested in other settings, this study aimed to pique interest and advance methodological considerations for more research on wage conditions in the health policy research workforce using an intersectoral and gender-based analysis lens.

## Data Availability

The data that support the findings of the study are available through Statistics Canada’s Research Data Centres but restrictions apply to the availability of these confidential data, which were used with permission for the current study, and so are not publicly available.
